# The Association of Falls with Instability: An Analysis of Perceptions and Expectations toward the Use of Fall Detection Devices Among Older Adults in Malaysia

**DOI:** 10.3389/fpubh.2021.612538

**Published:** 2021-02-12

**Authors:** Kawthar Abdul Rahman, Siti Anom Ahmad, Azura Che Soh, Asmidawati Ashari, Chikamune Wada, Alpha Agape Gopalai

**Affiliations:** ^1^Programme of Gerontechnology, Malaysian Research Institute on Ageing, Universiti Putra Malaysia, Serdang, Malaysia; ^2^Department of Electrical and Electronic Engineering, Faculty of Engineering, Universiti Putra Malaysia, Serdang, Malaysia; ^3^Department of Human Development and Family Studies, Faculty of Human Ecology, Universiti Putra Malaysia, Serdang, Malaysia; ^4^Graduate School of Life Science and System Engineering, Kyushu Institute of Technology, Kitakyushu, Japan; ^5^Advanced Engineering Platform, School of Engineering, Monash University Malaysia, Subang Jaya, Malaysia

**Keywords:** older adults, fall detection, fall prevention, instability, perception, assistive technology, gerontechnology

## Abstract

**Background:** Falls are a significant incident among older adults affecting one in every three individuals aged 65 and over. Fall risk increases with age and other factors, namely instability. Recent studies on the use of fall detection devices in the Malaysian community are scarce, despite the necessity to use them. Therefore, this study aimed to investigate the association between the prevalence of falls with instability. This study also presents a survey that explores older adults' perceptions and expectations toward fall detection devices.

**Methods:** A cross-sectional survey was conducted involving 336 community-dwelling older adults aged 50 years and older; based on randomly selected participants. Data were analyzed using quantitative descriptive analysis. Chi-square test was conducted to investigate the associations between self-reported falls with instability, demographic and walking characteristics. Additionally, older adults' perceptions and expectations concerning the use of fall detection devices in their daily lives were explored.

**Results:** The prevalence of falls was 28.9%, where one-quarter of older adults fell at least once in the past 6 months. Participants aged 70 years and older have a higher fall percentage than other groups. The prevalence of falls was significantly associated with instability, age, and walking characteristics. Around 70% of the participants reported having instability issues, of which over half of them fell at least once within 6 months. Almost 65% of the participants have a definite interest in using a fall detection device. Survey results revealed that the most expected features for a fall detection device include: user-friendly, followed by affordably priced, and accurate.

**Conclusions:** The prevalence of falls in community-dwelling older adults is significantly associated with instability. Positive perceptions and informative expectations will be used to develop an enhanced fall detection incorporating balance monitoring system. Our findings demonstrate the need to extend the fall detection device features aiming for fall prevention intervention.

## Introduction

Falls are rising globally with an estimated 646,000 yearly fatal falls, where death rates among older adults aged 60 years old and above are the highest, worldwide (1). Falls are reported as the second leading cause of accidental or unintentional injury deaths worldwide after traffic injuries, and the leading cause for non-transport unintentional injury deaths (1, 2). Globally, it is reported that ~28–35% of older adults over the age of 65 years are estimated to fall once or more frequently each year (1). Whereas, in Malaysia, according to the National Health and Morbidity Survey in 2018 for elderly health reported nearly 15% of older adults aged 60 years or older reported at least one fall in 12 months (3).

In line with the demographic transition and speed of aging, the older adults' population growth brought safety among older adults into perspective and is always associated with falls, risk of falls and the impacts of the incidents. Fall incidents are typically associated with mortality, morbidity, and higher nursing home admission rates causing pricey social and healthcare costs (4). This may result in fear of falling developed and can contribute to psychological conditions such as depression and voluntary activity avoidance (4–7). The different levels of functional capability are significantly determined by the health-related quality of life (6). Other common risks in addition to advancement in age include balance impairment, walking difficulties, lower body weakness, vitamin D deficiency, vision problems, foot pain, poor footwear and hazardous living environment (8, 9).

Identifying factors associated with falls incidents is vital in formulating fall prevention, aiming at reducing the occurrence and further complications. The specific objective of the present study was, therefore, to analyze the prevalence of falls in older adults and its association with instability along with several noteworthy variables. Previous studies that examined instability reported various findings, where Lamb et al. (10) and Hyndman and Ashburn (11) discovered that instability was a risk factor for falls, as opposed to findings of Jorgensen et al. (12). The more risk factors that a person has would elevate to higher chances of falls. Thus, one of the solutions to maintain physical functioning is the use of assistive technology. It has become a vital necessity to help those in need during their advanced years.

Previously, researchers have put various efforts in developing fall prevention technologies to enhance older adults' functional capability as well as patients with functional impairment (13–22). Use of technology in fall prevention is increasing due to the limited skilled caregivers, a slow response that could lead to fatality and also high cost incurred. As identified by Khosravi and Ghapanchi (16), switching from complete reliance on human assistance to technology is assumed to be the viable solution to alleviate the gap between the demand for aged care and supply. Habib et al. (16) in their literature review, concluded that a fall detection system is a device to assist older adults and their caregivers in avoiding the consequences of “long lie” periods by a proposed series of events; detecting fall, triggering notification alarms, sending messages and calling for help as soon as a fall occurs. The term “long lie” is identified as unwillingly remaining on the ground for an hour or more following a fall, whereby half of those who experienced it die within 6 months of falling (23–25). Concerning this matter, older adults who fell with no fatal injuries but remained on the floor for prolonged periods after the fall must be closely monitored as they are categorized as individuals with a high risk of falls (26).

Common in the industry, commercial fall detection device offers the basic function of detecting a fall and immediately alert caregivers or monitoring base unit to provide necessary actions depending on the person's condition. Unfortunately, the development of fall detection devices has been controversially reviewed by users and researchers, which leads to halting the promising technology to be commercialized. For example, robustness in terms of the sensors' sensitivity and accuracy contribute to false detection due to a variety of fast movements. Failure in detecting different types of falls caused the system to automatically cancel the alert if the sensors detect any movements after the fall, assuming the person is conscious and needs no further help (27). Most of the devices in the market are only limited to detecting falls. While the technology is mostly geared toward older adults prone to fall, instability is a complementary subject concerning fall prevention programs. Therefore, this study is the groundwork in proposing an extended version of fall detection incorporating a balance monitoring system as a fall prevention device to be implemented in the future.

Additionally, another objective of this study was to analyze the psychological part concerning older adults. The perceptions on the use of fall detection devices were investigated. The perception of fear, frustration, and unacceptance toward technological devices as well as the refusal to adopt technology were examined at the end of this study. The issue of privacy being violated or reluctant to reach for tailored training in using the new technological device were examined. Their expectations toward fall detection devices were documented to understand older adults' preferences in utilizing such devices. Williams et al. (28) verified that the essential functions of the future assistive technology device include emergency alarm systems and fall detection. However, despite the perks that the technology could offer, there are risks involving privacy, and confidentiality due to continuous monitoring of movement via sensors and Global Positioning System (GPS) hence become significant concerns that can hinder its successful implementation (28–30). However, Malaysia-based studies of older adult's perceptions, the needs or acceptance of assistive technology remain insufficient. There was no previous study in Malaysia addressing the older adults' perceptions and expectations toward the use of fall detection devices. The particular needs and preferences of older adults in the context of the development and use of fall detection systems have not been given considerable attention (31). Malaysia's vital challenge is planning and managing the aging society, especially in providing necessary alternative for effective assistance for them.

## Materials and Methods

### Study Design and Setting

A nationwide cross-sectional survey was conducted targeting community-dwelling older adults. The sampling technique of this study was randomized with convenient sampling. This observational study was designed to find: (a) the association between prevalence of falls with instability among older adults, (b) the association between prevalence of falls and instability with demographic and self-reported walking characteristics, and (c) the perceptions and expectations toward fall detection devices based on the response from structured questionnaires. Data collection was conducted from July 2019 to January 2020. The survey was conducted using a confidential self-administered questionnaire distributed to random sample of older adults stratified by age, gender, and geographic location; representing a population estimate of 10,994,000 older adults aged over 50 years. The targeted sample of 385 older adults was determined using the Finite Population Correction, and the response rate was 87.3% (336 respondents).

### Participants

A total of 336 community-dwelling older adults aged 50 years and older participated in the study. The only inclusion criterion for participants was age. Even though older adults in Malaysia are defined as individuals aged 60 years old, pre-elderlies aged 50 to 59 years old were included in the process of selecting the participants due to lower life expectancy compared to the Western Countries (32) and referring to the National Health and Morbidity Survey (3). Also, knowing the health conditions of the pre-elderly group is vital as early detection of one's condition that can make a difference later in life. The goal of the target population was to have a wide-ranging representative of older adults nationwide. Participants involved were from various states across Malaysia that are having high number of older population, with the highest participants were from Perak (*n* = 160), followed by Selangor (*n* = 96) and Kuala Lumpur (*n* = 44). Other participants were scattered around other states (Johor, Kedah, Kelantan, Negeri Sembilan, Pulau Pinang, Putrajaya, and Terengganu). We selected the state of Perak as the main target in accordance to the population projection by the Department of Statistics Malaysia. The aging population in Perak is expected to become the oldest amongst the elderly population in Malaysia by 2020 when that group reaches 397,400 (15.8%) of the estimated 2.6 million total population (33).

### Ethical Considerations

The surveyed respondents were informed of the research's purpose, mode of participation, benefits and confidentiality. All respondents understood the objective of this research and voluntarily signed consent forms prior to the interview. Participation was entirely voluntary, and data were conducted confidentially where names and addresses were not being used in the analysis of this study. All study procedures were reviewed and approved by the Ethics Committee for Research Involving Human Subjects Review Board (JKEUPM-2017-251).

### Study Variables/Measurements

The questionnaire consisted of two self-administered parts on fall risk assessment following completion of their demography details; (i) Falls assessment and perceived walking stability and (ii) Experience using a fall detection device, perceptions, and expectations toward the use of a fall detection device. Participants were enlightend earlier on the fall definition, which was defined as an unexpected event in which the individual comes to rest on the ground, floor or lower level (1). As for the fall detection device, the basic mechanism was explained and defined as a wearable device. The advantage of using the fall detection device was enlightened. An example of an existing fall detection device was displayed to the participants upon answering the questionnaire.

In this paper, only the critical measurements mentioned below were extracted and examined to determine the contributing factors associated with fall incidents.

1. The primary outcome for this study is the prevalence of falls in the past six months, that was ascertained by questioning, “Did you experience any fall in the past six months?” prior to answering the questionnaire.
2. Walking stability was derived from a close-ended question of “Do you feel unbalance/ unstable while walking/ moving to places.” Meanwhile, the answers to multiple response questions regarding their walking characteristics such as “Walking with a bit stooped, walking with shuffle foot, difficulty rising from a chair, cannot walk without assistance, not having such problems,” and “Do you need a walking aid to assist you in walking/moving?” are then referred to ensure the consistency of answers. Respondents were considered as having a mobility limitation if they indicate a need for any of the listed ambulatory aids (wheelchair, cane, crutches, and/or clutching onto the furniture or anything around them to move from one place to another).
3. Perception toward fall detection devices was attained from three questions which were “What makes you refuse to use a fall detection device?”, “Do you think the use of this fall detection device can increase your safety and reduce the risk of falls?”, and “If you have been offered to test a fall detection device, are you interested to try it on?”.
4. Lastly, participants were required to answer an open-ended question “What is your expectation of a fall detection device if you are to use one?”. The answers were summarized and grouped according to the frequencies, resulting in a list of older adults' expected features in a fall detection device.

### Quantitative Data Collection and Analysis

The design of the questionnaire was adapted from the Johns Hopkins Fall Risk Assessment Tool (JHFRAT) for Home Health Care, Morse Fall Scale and Outpatient Falls Questionnaire by Abujudeh et al. (34), modified to suit the Malaysian population. Frequency and percentage were used for descriptive analysis based on the quantitative data. The strength of association was analyzed using Chi-square test for inferential statistics with 95% confidence interval. The three computed associations were: (i) between falls with instability, (ii) between falls and instability with demographic characteristics, and (iii) between falls and instability with walking characteristics. *P*-values were based on two-sided tests and were considered statistically significant at *p* < 0.05. All analyses were conducted using SPSS software (SPSS Inc., version 22.0).

## Results

Quantitative results were based on data collected using questionnaires, in which 336 respondents have successfully participated in the survey. There are three distinct sections of analysis: (i) Association between falls and instability with demographic and walking characteristics, (ii) Association between falls with instability, and (iii) Perceptions and expectations toward fall detection devices. The prevalence of falls and perceived instability among respondents was compared across gender, age groups and living arrangements. Further analysis of walking characteristics among the respondents was done with regards to the use of ambulatory aids and perceived gait problems.

### Association Between Falls and Instability With Demographic and Walking Characteristics

The average age of the respondents was 63.6 years (SD: 7.2 years), with 83 individuals were <60 years old, and eight individuals above 80 years old. The majority were females (83.3%), and 85.7% of respondents co-reside with family members including spouse, children and other family members. In total, *n* = 336 respondents successfully provided data on falls and stability.

Table 1 shows the association between falls with demographic and walking characteristics. The prevalence of falling in 6 months prior to the survey was 28.9% (*n* = 336), with 24.1% reported to fall once or twice. Men reported a 3.9% higher prevalence of falls than women as the frequency for falling three or more times was higher among male respondents. The association between the prevalence of falls with age was statistically significant as falls increases with age. Gender and living arrangement have no significant association with the prevalence of falls, although male respondents and those who lived with children showed higher prevalence compared to other groups.

**Table 1:** The association between falls with demographic and walking characteristics.

**Variables**	**Total (*n*, %)**	**Falls in 6 months (*****n*****, %)**			**X^**2**^**	***p*-value**
		**1–2**	**3 or more**	**None**		
**Total respondents (*****n*****, %)**	336 (100)	81 (24.1)	16 (4.8)	239 (71.1)	-	-
*Demographic characteristics*						
**Gender**						
Male	56 (16.7)	12 (21.4)	6 (10.7)	38 (67.9)	5.301	0.071
Female	280 (83.3)	69 (24.6)	10 (3.6)	201 (71.8)		
**Age (Years)**						
50–59	83 (24.7)	19 (22.9)	4 (4.8)	60 (72.3)	13.922	^*^
60–69	181 (53.9)	39 (21.6)	4 (2.2)	138 (76.2)		
70–79	64 (19)	21 (32.8)	7 (10.9)	36 (56.3)		
>80	8 (2.4)	2 (25)	1 (12.5)	5 (62.5)		
**Living arrangement**						
Alone	48 (14.3)	9 (18.8)	4 (8.3)	35 (72.9)	5.517	0.479
Spouse	121 (36)	34 (28.1)	3 (2.5)	84 (69.4)		
Children	62 (18.4)	17 (27.4)	3 (4.9)	42 (67.7)		
Family member	105 (31.3)	21 (20)	6 (5.7)	78 (74.3)		
*Walking characteristics*						
**Ambulatory aid type**						
Wheelchair	4 (1.2)	2 (50)	1 (25)	1 (25)	18.378	^***^
Cane	21 (6.3)	9 (42.9)	4 (19)	8 (38.1)		
Crutches	3 (0.9)	1 (33.3)		2 (66.7)		
Clutching onto the furniture	21 (6.3)	7 (33.3)	2 (9.5)	12 (57.2)		
None	287 (85.3)	64 (22.3)	8 (2.8)	215 (74.9)		
**Perceived gait problem**						
Walking with a bit stooped	25 (7.4)	5 (20)	4 (16)	16 (64)	14.504	^**^
Walking with shuffle foot	24 (7.1)	12 (50)	4 (16.7)	8 (33.3)		
Difficulty rising from a chair	57 (17)	19 (33.3)	3 (5.3)	35 (61.4)		
Cannot walk without assistance	8 (2.4)	2 (25)	2 (25)	4 (50)		
Not having such problems	235 (69.9)	47 (20)	7 (3)	181 (77)		

* *p-value < 0.05*,

** *p-value < 0.01*,

*** *p-value < 0.001*.

Furthermore, when comparing across the variables related to walking characteristics, one out of seven respondents were using ambulatory aids to help them walking or moving around. There was a statistically high and significant association between falls and using ambulatory aids (*p* < 0.001). Respondents who depend on ambulatory aids, especially wheelchair and cane, were more likely to fall as the frequency of falls was higher.

Respondents perceived to have the listed gait problems tend to fall more, in which 101 reported walking difficulties, correspondingly having significant association with falls. The frequency of falls across respondents walking with shuffle foot was the highest, where almost 70% fell at least once in 6 months. The ones without gait problems also reported 23% falls incidents among 235 respondents. Indeed, not only respondents with gait problems were having instability issues.

A similar analysis was made across demographic and walking characteristics associated with perceived instability as presented in Table 2. The results were based on respondents' self-reported walking stability. Around 22% of the respondents perceived instability when moving from one place to another. Around one-third of both males and females reported having instability issues, but no significant association was found between gender and perceived instability. Similar to the prevalence of falls, there was a significant association between age and perceived instability as the frequency increases with age. However, no association between living arrangements and perceived instability was found, but respondents who lived alone reported higher instability issues percentage.

**Table 2:** The association between instability with demographic and walking characteristics.

**Variables**	**Perceived instability (*****n*****, %)**		**X^**2**^**	***p*-value**
	**Yes**	**No**		
**Total respondents (*****n*****, %)**	72 (21.4)	264 (78.6)	-	-
*Demographic characteristics*				
**Gender**				
Male	15 (26.8)	41 (73.2)	1.145	0.285
Female	57 (20.4)	223 (79.6)		
**Age (Years)**				
50–59	17 (20.5)	66 (79.5)	17.374	^**^
60–69	31 (17.1)	150 (82.9)		
70–79	18 (28.1)	46 (71.9)		
>80	6 (75)	2 (25)		
**Living arrangement**				
Alone	13 (27.1)	35 (72.9)	3.794	0.285
Spouse	28 (23.1)	93 (76.9)		
Children	15 (24.2)	47 (75.8)		
Family member	16 (15.2)	89 (84.8)		
*Walking characteristics*				
**Ambulatory aid type**				
Wheelchair	4 (100)		117.879	^***^
Cane	21 (100)			
Crutches	2 (66.7)	1 (33.3)		
Clutching onto the furniture	16 (76.2)	5 (23.8)		
None	31 (10.8)	256 (89.2)		
**Perceived gait problem**				
Walking with a bit stooped	12 (48)	13 (52)	88.03	^***^
Walking with shuffle foot	17 (70.8)	7 (29.2)		
Difficulty rising from a chair	29 (50.9)	28 (49.1)		
Cannot walk without assistance	7 (87.5)	1 (12.5)		
Not having such problems	18 (7.7)	217 (92.3)		

** *p-value < 0.01*,

*** *p-value < 0.001*.

The association between perceived instability with the use of ambulatory aids and perceived gait problems were highly significant. All wheelchair and cane users were found to have instability issues. Almost 90% of the respondents who cannot walk without assistance also reported having instability while moving between places. Also, it is noted that 31 respondents with instability issues did not opt to use ambulatory aids in their daily lives.

### Association Between Falls and Instability

Table 3 shows a statistically high and significant association between falls and perceived instability. The prevalence of falls among the respondents who perceived instability was 47.2%. Among respondents with instability issues, over one-third reported falling at least once in 6 months prior to the survey, and 12.5% reported falling three times or more. Moreover, the percentage of respondents with perceived instability issues reported to fall was directly proportional with age; 10.8, 6.6, 15.6, and 37.5% (sequence follows the age group in Table 1).

**Table 3:** The association between falls with instability.

**Falls in 6 months (*n*, %)**	**Perceived instability (*****n*****, %)**		**X^**2**^**	***p*-value**
	**Yes**	**No**		
1–2	25 (34.7)	56 (21.2)		
3 or more	9 (12.5)	7 (2.7)	20.145	^***^
None	38 (52.8)	201 (76.1)		

*** *p-value < 0.001*.

### Perceptions and Expectations Toward Fall Detection Devices

For this section, the respondents were initially asked whether have known or heard of a fall detection device, and 91.7% of them answered No. Nonetheless, respondents who answered Yes, stated that they refuse to use the device due to these reasons; they have never experienced falls (*n* = 17), no information on how or where to purchase the device (*n* = 8), and because the device is expensive (*n* = 2).

In addition, an equally important finding is that one respondent has experienced using a fall detection device. The respondent stopped using the device after a few months because the wearable device was mentioned to be uncomfortable. However, the device was believed to provide safety and helpful in reducing the risk of falls.

Almost 99% of 336 respondents agreed that fall detection devices could help to increase safety and reduce the risk of falls, and 64% have a definite interest in using a fall detection device. In comparison, 33% answered Maybe. Referring to Table 4, of those who said yes, their history of falls showed higher prevalence compared to those who were not interested. Similarly, respondents perceived to have instability showed a positive interest in using the fall detection device. The percentage of interest was surprisingly high among older respondents and those living with children.

**Table 4:** Interest in using fall detection devices in relation to the prevalence of falls, perceived instability and demographic characteristics among the respondents.

**Interest in using a fall detection device**	**Yes (*n*, %)**	**Maybe (*n*, %)**	**Not interested (*n*, %)**
**Falls in 6 months**			
1–2	59 (72.8)	22 (27.2)	
3 more	12 (75)	4 (25)	
None	144 (60.2)	85 (35.6)	10 (4.2)
**Perceived instability**			
Yes	57 (79.2)	14 (19.4)	1 (1.4)
No	158 (59.9)	97 (36.7)	9 (3.4)
**Sex**			
Male	34 (60.7)	21 (37.5)	1 (1.8)
Female	181 (64.7)	90 (32.1)	9 (3.2)
**Age (Years)**			
50–59	36 (43.4)	45 (54.2)	2 (2.4)
60–69	122 (67.4)	53 (29.3)	6 (3.3)
70–79	52 (81.3)	10 (15.6)	2 (3.1)
>80	5 (62.5)	3 (37.5)	
**Living arrangement**			
Alone	32 (66.7)	14 (29.2)	2 (4.1)
Spouse	81 (66.9)	37 (30.6)	3 (2.5)
Children	46 (74.2)	14 (22.6)	2 (3.2)
Family member	56 (53.3)	46 (43.8)	3 (2.9)

**Figure 1:**
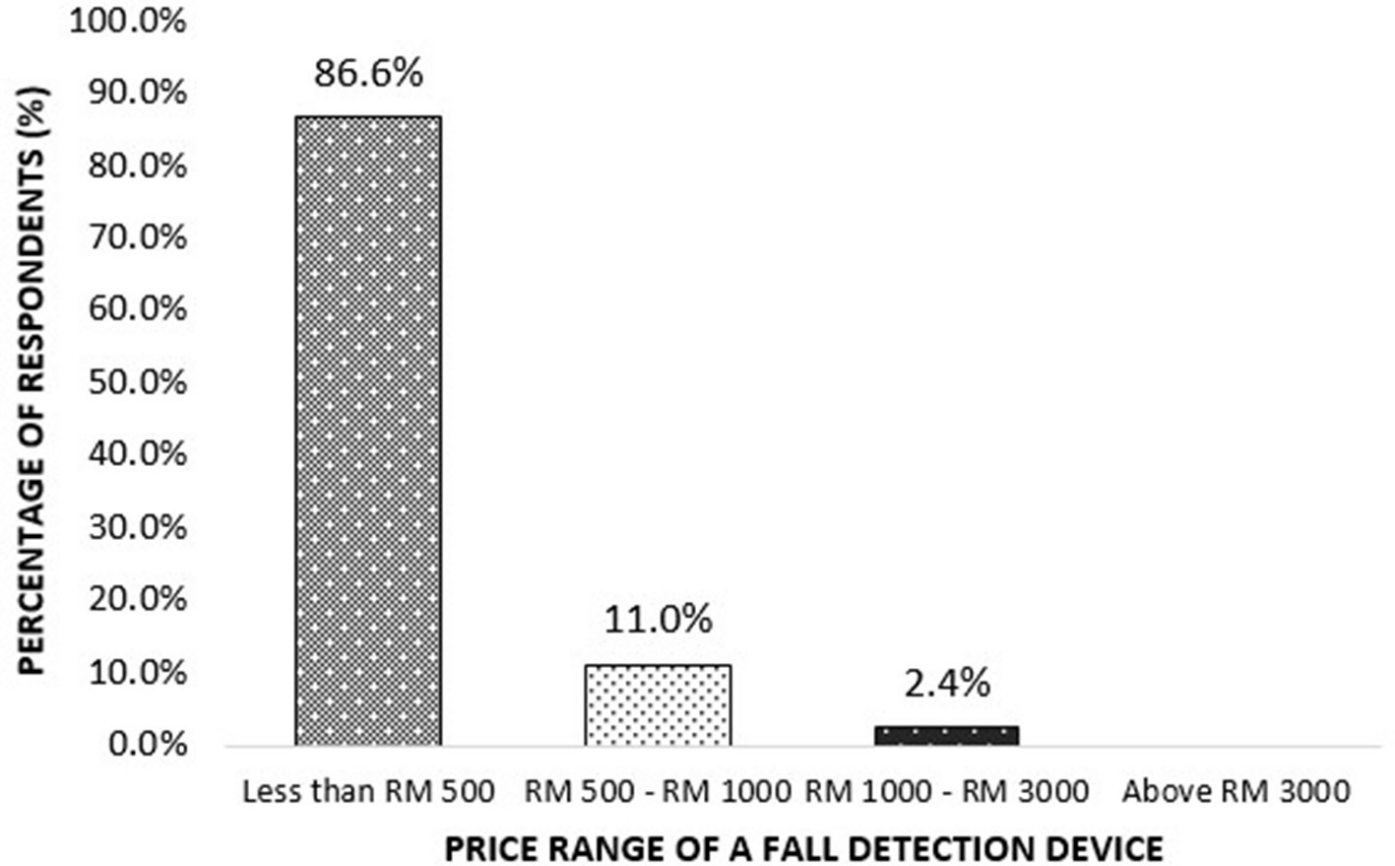
Expected price range of a fall detection device by the respondents.

Price plays an important role when purchasing a device, especially among older adults who are generally technologically illiterate. When asked about the expected price range of a fall detection device, most of them answered the device should be < RM500, and only 2.4% of them selected RM1000–3000 (Figure 1).

**Figure 2:**
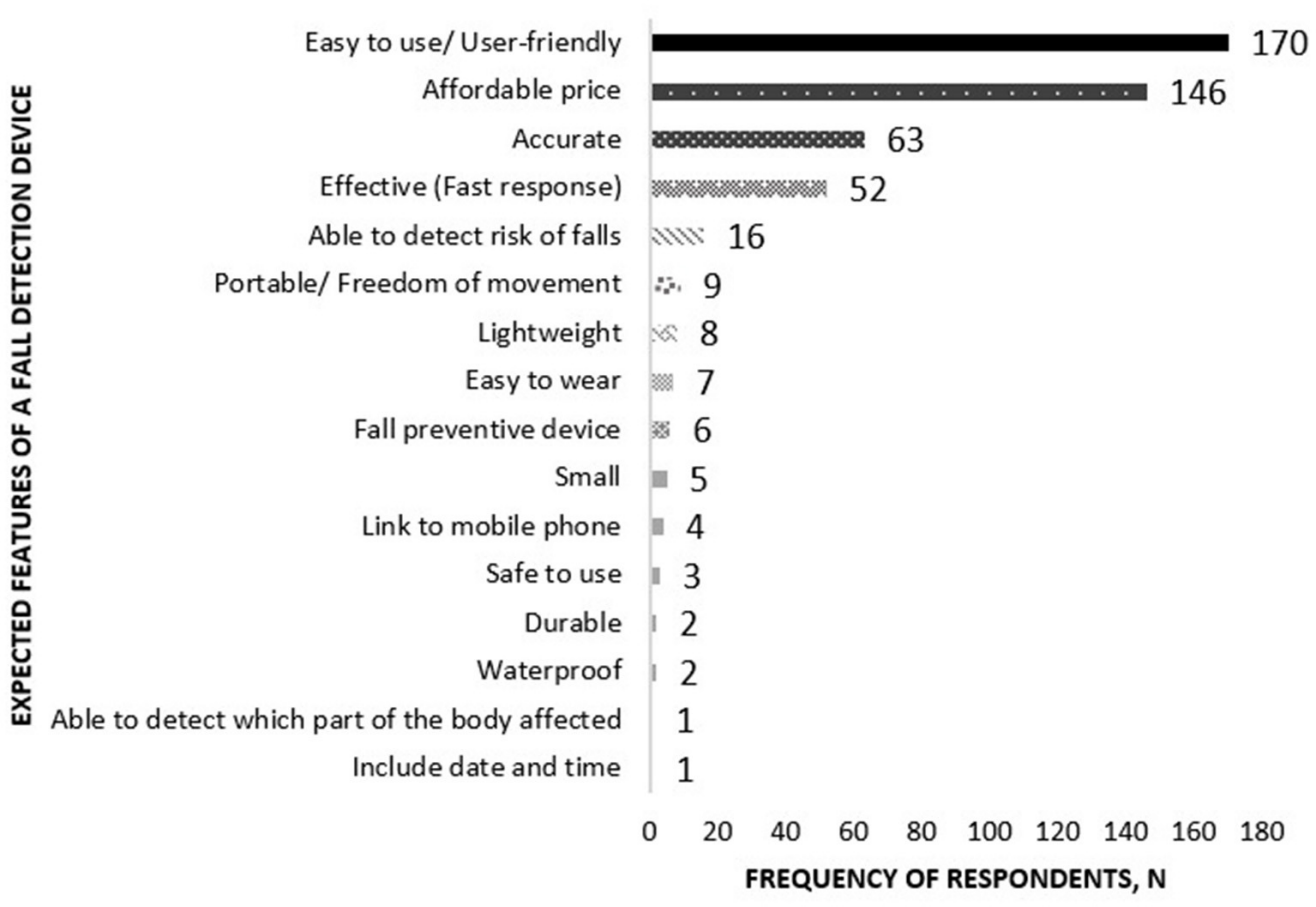
Expected features of a fall detection device by the respondents.

The last question was open-ended, where expected features being anticipated in a fall detection device were inquired. The most answered feature was easy to use or user-friendly (*n* = 170) followed by affordable price (*n* = 146), accurate (*n* = 63), and effective in terms of fast response (*n* = 52). The rest of the answers are illustrated in Figure 2.

## Discussion

This study's results highlight the association between falls with instability and their association across demographic and walking characteristics among a sample of older adults aged 50 years and above. Also, their perceptions toward the use of fall detection devices were investigated in which safety and reducing the risk of falls are the two main concerns, along with the expected features of fall detection devices. These findings identify potential enhanced features to be incorporated in a fall detection device by studying the older population's fall risk profile and may influence intervention strategies aimed at preventing falls.

Almost half of the respondents who reported falls in the past 6 months perceived they suffered instability while walking or moving to places. Since the *p*-value is less than significant level 0.05, we can conclude that there is an association between falls and instability among older adults in Malaysia (X^2^ = 20.145 and *p*-value < 0.001). There was an increasing trend across age group in terms of the prevalence of falls and perceived instability. The results support previous literature findings that gait difficulties or instability were associated with falls in older adults (7, 26, 35–40). Older adults tend to define a fall as a loss of balance, while health care professionals commonly address the consequence of falling, including injury and reduced quality of life (41). Hatch et al. (39) suggested that older adults who have concerns about their balance may encounter actual balance deficits. This explains why the respondents with instability issues in this study reported a high number of falls occurrences, at the same time increasing the risk of falls. Covinsky et al. (42) and Muir et al. (9) emphasized their findings that the self-reported dizziness or instability is associated with increased fall risk.

The prevalence of falls in the last 6 months prior to the survey was 28.9% (*n* = 336), consistent with a study done in institutional settings in Kuala Lumpur, with 30% (*n* = 50) prevalence (43) and 27.3% (*n* = 516) with history of falls in Melaka (44). In another study conducted in Brazil, with 400 participants aged 60 years or older, the prevalence of falls was 35.3% (45). The result was higher compared to other several studies reporting the prevalence of falls in the preceding 12 months; 4.07% (*n* = 811) by a study among community-dwelling in Perak (46), 14.1% (*n* = 3,969) from the National Health and Morbidity Survey 2018 (3), and 17.6% (*n* = 1,372) among community-dwelling older adults aged 40 and above in Germany (47). Also, a recent study of older adults living in four regions across the United States reported 18.8% (*n* = 878) prevalence in the previous 6 months (13).

The risk of falling is increasing with age as the findings found that increasing age was statistically significant, where the age group of 70 years and above were more likely to fall, mainly three or more times in the past 6 months. The findings are similar to those found in studies analyzed that advanced age is associated with a higher percentage of falls (8, 36, 45, 48–50). In contrast, Rizawati and Mas Ayu (44) found that fall occurrence was the highest in the younger group of 60 to 70 years, which accounted for 59.6% of total falls compared to age more than 70 years old (32.7%) during the past 12 months.

Although living arrangement has no significant association with the prevalence of falls and perceived instability, the ones who need serious attention are older adults who live alone, as there were 13 cases of falls reported in 6 months. Family members or designated community could remotely monitor from elsewhere if they were to use any fall detection devices to avoid long lie situation if a fall occurs and subsequently would provide immediate assistance.

More males respondents were experiencing instability. Older age group and those living alone are prone to have risk of falls, in consistence with the known relationship between falls and instability. The analysis revealed that regardless of gender and living arrangements, there were still incidents of falls and instability among the respondents.

Reliance on ambulatory aids to move around in daily activities is identified as mobility limitation among older adults. Over a quarter of those who did not use ambulatory aids reported fall incidents. The results revealed that respondents who relied on ambulatory aids tend to fall more, contributing to the increased risk of falls among older adults. Thus, it necessitates fall detection device. The present study is consistent with the findings from previous studies reporting that respondents with a history of falls were more likely to use an assistive device (37, 51), but contradicting with the findings from Harris and colleagues (35). Also, Bogle and Newton (52) identified that the use of an assistive device was a strong predictor of performance on the Berg balance test.

Older adults are encouraged to depend on assistive devices during their advanced years to avoid accidents. The association between using assistive devices for ambulation and falls concludes that older adults require assistance from caregivers or family members, suggesting that additional training with the appropriate assistive device is necessary. The impaired balance resulting from the aging process among the respondents may require the use of ambulatory aids, as deduced from the findings that all types of ambulatory aids used were related to almost 90% of the respondents with instability issues.

The present study's findings agree with other studies that concluded respondents who fall were more likely to have gait problems; identified to be the factors in fall incidence and risk (8, 37, 45, 49, 53–56). Tinetti et al. (26) also revealed balance and gait tests, including difficulty rising from a chair, in which half of the respondents from this study reported having gait problems, to be a useful predictor in identifying recurrent fallers. Furthermore, investigating the association of instability and gait problems led to the term of fear of falling. Hatch et al. (29) stated that fear of falling was a factor of balance confidence concerning the impaired balance due to these balance limitations.

Preparing for the aging population comes with challenges. One of them is dealing with older adults themselves. Their perceptions and acceptance toward technology with rapid innovation in this day and age may affect the percentage of adoption and usage. For example, most of the respondents in this study have never heard of the fall detection device, though it is widely used in developed countries. It is unfortunate to say that older adults in Malaysia have yet to be well-literated with advanced technology. This may be because of decreasing cognitive ability making it hard to learn and use a new device with new features and buttons. Based on the survey results, 28 of the respondents who knew about the device have stated the reasons for refusal to use the fall detection device. Most stated they have not fallen before resulting in no intent of purchasing or trying to use the device. In future, a longer survey period and broader targeted samples with experience using a fall detection device may contribute to more meaningful analysis for older adults population in Malaysia.

In reality, older adults are interested in using a technological device that can assist them in their daily lives, as proven in Table 4. Majority of the respondents were interested in using a fall detection devices, provided they are tailored with training and assistance. Consistent with Wong (57), new technology adoption among older adults (in the study refers to mobile phone) took much longer time although it has positively changed from resistance to acceptance. In order to expedite the technology adoption, the barriers must be investigated and overcame. As identified by several studies, barriers include lacking in these matters: the knowledge of key elements of the program, the expertise to train programs concerning fall preventions, the confidence of using advanced technology, insufficient knowledge of target group, funding, public awareness, and marketing campaign regarding gerontechnology programs (18, 58–60).

A previous study agrees with the finding that older adults tend to assume that the device is expensive; therefore, they tend not to use any technological advanced device at all (61). This explains why 86.6% of the respondents chose the expected affordable and suitable price for the fall detection device to be the lowest (< RM500).

Findings also showed that most of the respondents and their community lacked information and awareness on the range of available assistive devices and other available devices that offer a quality life in their advanced years. Referring to Chen and Chan (62), the older adults' health capabilities and functional capacities affect the utilization of gerontechnology. Simultaneously, its usage is driven by outcome expectations and peer recommendation, supported by facilitators. Correspondingly, their study proved that training was the primary factor in facilitating technology use, and it will increase the chance of accepting and utilizating innovative technology (62). In-line with previous studies, training courses or workshops could boost older adult's self-confidence, stimulate positive attitudes, and surge in intention to use technology (63, 64). As such, The Cycle of Technology Acquirement by Independent-Living Seniors (C-TAILS) introduced by Peek et al. (65), older adults' problems or status quo must be investigated first before proposing new technology to them. Then, enabling mechanisms would be triggered as personal and situational moderating factors, influencing the technology acquirement, which then will be depicted by their actual experiences with the newly acquired technology.

The assessment highlights the importance of inquiring older adults' expectations regarding a future fall detection device's features. The findings can be applied as a guide strategies in designing the device and engaging the older population in preventing falls in the future.

## Limitations

The outcomes of this study have some limitations. Participants were randomly selected at convenience with the aim to have a wide-ranging older adult population. However, only healthy community-dwelling was selected due to limited access and a short data collection period. As they were recruited by convenience sampling, it cannot represent the general population and are prone to selection bias.

Secondly, incidents of falls and instability were assessed by self-report, which could lead to recall bias. As most retrospective research, the false recall may have created inaccuracies in reporting the fall history retrospectively over 6 months. The actual frequencies of falls would be greater or lesser than 28.9%. Additionally, the respondents' functional condition may not be the same at the time of the fall incidents while the respondents were evaluated.

Thirdly, there was no further assessment of falls-related factors and instability characteristics. Examples of assessments to develop more meaningful analysis are gait patterns, types of injuries caused by falls and symptoms or diseases that could cause falls (e.g., impaired vision, dizziness, vertigo, dementia, depression). Additionally, further studies on older adults' reactions to maintain balance in a time of perturbation are highly suggested.

Fourthly, findings represent by cross-sectional associations cannot be used in determining the cause. Further studies to address the causal relationship of falls, instability, gait impairments, and mobility limitations are needed.

Although the present study investigated the older adult's perceptions and the use of technology, the findings are based on cross-sectional studies that did not take into account user experiences.

## Conclusion

The prevalence of falls and the percentage of perceived instability conditions increases with time as the older population grows. The analysis showed that instability affects the occurrence of falls among older adults. The occurrences could be reduced with proper monitoring. The demand continues for fall prevention intervention in detection, as well as awareness and familiarization among this population.

As for the research approach, the questionnaire has been proven to be quite successful in analyzing the older adult's perception and expectation toward the use of fall detection devices in Malaysia. The questionnaire results identified a fall detection device's preferred design for older adults. In the field of innovation design for older adults, it is always important to mind our designs to be in tandem with aging competency to avoid the perception of fear, irritation, and refusal to use the technological device. Thus, the designed technology must be calibrated and developed with them instead of for them. Meanwhile, encouraging this non-users to adopt technology requires removing all barriers at personal, technological and environmental levels to ensure its effectiveness. With all variables considered, it can be concluded that the need to extend the fall detection device incorporating a balance monitoring system is highly beneficial for fall prevention intervention.

Although several variables distinguished between those who fell and those who did not, the relationship may only be an association, not causal; may vary between individuals, similar to perceived instability conditions. For example, having gait problems may cause an older adult to develop a fear of falling, loss of stability, and consequently tend to fall. Alternatively, an older adult may not have such problems and may fall, both because of the nature of aging.

The primary purpose of studying gerontechnology is to improve the quality of life among older adults. The proposed device offers older adults freedom and boosts their confidence whereby they can do daily activities without fear, hence promoting active aging. The more active an older adult is, the lesser potential of falling will it be. Technology is one of the approaches which can support older adults in their daily lives, in addition to enhancing their comfort and safety at home.

Henceforward, the well-being of older adults should be taken into consideration from now on. An enhanced fall prevention device is expected to assist caregivers or family members as real-time monitoring provides immediate alert, promoting peace of mind and reducing the burden. Correspondingly, physical therapists and clinicians can clearly understand when the person falls and circumstances surrounding it, allowing for better treatment. Finally, more profound findings and more psychological analysis are needed, which can be tailored to older user's needs and perceived to be senior-friendly and more accurate.

## Data Availability Statement

The original contributions generated in the study are included in the article/supplementary material, further inquiries can be directed to the corresponding author.

## Ethics Statement

The studies involving human participants were reviewed and approved by Ethics Committee for Research Involving Human Subjects (JKEUPM), Universiti Putra Malaysia. The patients/participants provided their written informed consent to participate in this study.

## Author Contributions

KA, SA, and CW designed the whole framework and methodology. KA conducted data collection, data analysis, and manuscript preparation. All authors were involved in the methodology development, read, review and proofread the final manuscript.

## Conflict of Interest

The authors declare that the research was conducted in the absence of any commercial or financial relationships that could be construed as a potential conflict of interest.

## References

[1] World Health Organization. WHO Global Report on Falls Prevention in Older Age. WHO Press, World Health Organization (2007). Available online at: http://www.who.int/ageing/publications/Falls_prevention7March.pdf (accessed December 11, 2018).

[2] Norton R Ahuja RB Hoe C Hyder AA Ivers R Keay L. Nontransport Unintentional Injuries. In: Mock CN Nugent R Kobusingye O Smith KR editors. 7th ed. Washington, DC: International Bank for Reconstruction and Development/The World Bank (2017). p. 55–70. 30212114

[3] National Institutes of Health (NIH) M of HM. National Health and Morbidity Survey 2018: Elderly Health. Volume Two: Elderly Health Findings. Vol. 2, Institute for Public Health, National Institutes of Health (NIH), Ministry of Health, Malaysia (2018). Available online at: http://www.iku.gov.my/nhms-2018 (accessed November 05, 2019).

[4] Scheffer AC Schuurmans MJ Van dijk N Van der hooft T De rooij SE. Fear of falling: measurement strategy, prevalence, risk factors and consequences among older persons. Age Ageing. (2008) 37:19–24. 10.1093/ageing/afm169 18194967

[5] Cumming RG Salkeld G Thomas M Szonyi G. Prospective study of the impact of fear of falling on activities of daily living, SF-36 scores, and nursing home admission. J Gerontol Series A Biol Sci Med Sci. (2000) 55:299–305. 10.1093/gerona/55.5.M299 10819321

[6] Suzuki M Ohyama N Yamada K Kanamori M. The relationship between fear of falling, activities of daily living and quality of life among elderly individuals. Nurs Health Sci. (2002) 4:155–61. 10.1046/j.1442-2018.2002.00123.x 12406202

[7] Belgen B Beninato M Sullivan PE Narielwalla K. The association of balance capacity and falls self-efficacy with history of falling in community-dwelling people with chronic stroke. Arch Phys Med Rehabil. (2006) 87:554–61. 10.1016/j.apmr.2005.12.027 16571397

[8] Ambrose AF Paul G Hausdorff JM. Risk factors for falls among older adults: a review of the literature. Maturitas. (2013) 75:51–61. 10.1016/j.maturitas.2013.02.009 23523272

[9] Muir SW Berg K Chesworth B Klar N Speechley M. Balance impairment as a risk factor for falls in community-dwelling older adults who are high functioning: a prospective study. Phys Ther. (2010) 90:338–47. 10.2522/ptj.20090163 20056721

[10] Lamb SE Ferrucci L Volapto S Fried LP Guralnik JM. Risk factors for falling in home-dwelling older women with stroke: the women's health and aging study. Stroke. (2003) 34:494–500. 10.1161/01.STR.0000053444.00582.B7 12574566

[11] Hyndman D Ashburn A Stack E. Fall events among people with stroke living in the community: circumstances of falls and characteristics of fallers. Arch Phys Med Rehabil. (2002) 83:165–70. 10.1053/apmr.2002.28030 11833018

[12] Jørgensen L Engstad T Jacobsen BK. Higher incidence of falls in long-term stroke survivors than in population controls: depressive symptoms predict falls after stroke. Stroke. (2002) 33:542–7. 10.1161/hs0202.102375 11823667

[13] Frieson CW Tan MP Ory MG Smith ML. Evidence-based practices to reduce falls and fall-related injuries among older adults. Front Public Health. (2018) 6:222. 10.3389/fpubh.2018.00222 30186826 PMC6110876

[14] Hamm J Money AG Atwal A Paraskevopoulos I. Fall prevention intervention technologies: a conceptual framework and survey of the state of the art. J Biomed Inform. (2016) 59:319–45. 10.1016/j.jbi.2015.12.013 26773345

[15] Mahoney JE Clemson L Schlotthauer A Mack KA Shea T Gobel V. Modified Delphi consensus to suggest key elements of stepping on falls prevention program. Front Public Health. (2017) 5:21. 10.3389/fpubh.2017.00021 28265557 PMC5317011

[16] Habib MA Mokhtar MS Kamaruzzaman SB Lim KS Pin TM Ibrahim F. Smartphone-based solutions for fall detection and prevention: challenges and open issues. Sensors (Switzerland). (2014) 14:7181–208. 10.3390/s140407181 24759116 PMC4029687

[17] Chaccour K Darazi R El Hassani AH Andres E. From fall detection to fall prevention: a generic classification of fall-related systems. IEEE Sens J. (2016) 17:812–22. 10.1109/JSEN.2016.2628099

[18] Day L Trotter MJ Donaldson A Hill KD Finch CF. Key factors influencing implementation of falls prevention exercise programs in the community. J Aging Phys Act. (2016) 24:45–52. 10.1123/japa.2014-0143 25838262

[19] Colon LNV DeLaHoz YS Labrador MA. Human fall detection with smartphones. In: 2014 IEEE Latin-America Conference on Communications; 2014 Nov 5. LATINCOM: IEEE (2014). p. 1–7.

[20] Nizam Y Mohd MN Jamil M. Development of a user-adaptable human fall detection based on fall risk levels using depth sensor. Sensors. (2018) 18:2260. 10.3390/s18072260 30011823 PMC6069164

[21] Soewito B. Medical alert system using fall detection algorithm on smartphone. Int J Softw Eng Appl. (2015) 9:67–86. 10.14257/ijseia.2015.9.1.06

[22] Renner R Behnke S. Instability detection and fall avoidance for a humanoid using attitude sensors and reflexes. In: 2006 IEEE/RSJ International Conference on Intelligent Robots and Systems; 2006 Oct 9. Beijing: IEEE (2006). p. 2967–73.

[23] Fleming J Brayne C. Inability to get up after falling, subsequent time on floor, and summoning help: prospective cohort study in people over 90. BMJ. (2008) 337:a2227. 10.1136/bmj.a2227 19015185 PMC2590903

[24] Bisson EJ Peterson EW Finlayson M. Delayed initial recovery and long lie after a fall among middle-aged and older people with multiple sclerosis. Arch Phys Med Rehabil. (2015) 96:1499–505. 10.1016/j.apmr.2015.04.012 25933915

[25] Simpson PM Bendall JC Tiedemann A Lord SR Close JCT. Epidemiology of emergency medical service responses to older people who have fallen: a prospective cohort study. Prehospital Emerg Care. (2014) 18:185–94. 10.3109/10903127.2013.856504 24401155

[26] Tinetti ME Speechley M Ginter SF. Risk factors for falls among elderly persons living in the community. N Engl J Med. (1988) 319:1701–7. 10.1056/NEJM198812293192604 3205267

[27] Wang Z Ramamoorthy V Gal U Guez A. Possible life saver: a review on human fall detection technology. Robotics. (2020) 9:55. 10.3390/robotics9030055

[28] Williams V McCrindle R Victor C. Older people's perceptions of assistive technology—an exploratory pan-european study. J Integr Care. (2010) 18:38–44. 10.5042/jic.2010.0086

[29] Hensel BK Demiris G Courtney KL. Defining obtrusiveness in home telehealth technologies: a conceptual framework. J Am Med Inform Assoc. (2006) 13:428–31. 10.1197/jamia.M2026 16622166 PMC1513674

[30] Percival J Hanson J. Big brother or brave new world? Telecare and its implications for older people's independence and social inclusion. Crit Soc Policy. (2006) 26:888–909. 10.1177/0261018306068480

[31] Thilo FJS Hürlimann B Hahn S Bilger S Schols JMGA Halfens RJG. Involvement of older people in the development of fall detection systems: a scoping review. BMC Geriatrics. (2016) 16:42. 10.1186/s12877-016-0216-3 26869259 PMC4750302

[32] Ashari A. Fall Risk Assessment and Effectiveness of Home Based Exercise on Balance and Functional Mobility among Malaysian Adult Aged 50 years and above (dissertation thesis). Western Australia, Australia (2017).

[33] Department of Statistics Malaysia. Data from: Population Quick Info. (2020). Available online at: http://pqi.stats.gov.my/result.php?token=62293733fca4067e6708650c211e88ce (accessed June 2, 2019).

[34] Abujudeh HH Aran S Besheli LD Miguel K Halpern E Thrall JH. Outpatient falls prevention program outcome: an increase, a plateau, and a decrease in incident reports. Am J Roentgenol. (2014) 203:620–6. 10.2214/AJR.13.11982 25148166

[35] Harris JE Eng JJ Marigold DS Tokuno CD Louis CL. Relationship of balance and mobility to fall incidence in people with chronic stroke. Phys Ther. (2005) 85:150–8. 10.1093/ptj/85.2.150 15679466

[36] Campbell AJ Borrie MJ Spears GF. Risk factors for falls in a community-based prospective study of people of 70 years and older. J Gerontol. (1989) 44:M112–7. 10.1093/geronj/44.4.m112 2738307

[37] Lipsitz LA Johnsson PV Kelley MM Koestner JS. Causes and correlates of recurrent falls in ambulatory frail elderly. J Gerontol. (1991) 46:M114. 10.1093/geronj/46.4.M114 2071832

[38] Maki BE McIlroy WE. Postural control in the older adult. Clin Geriatr Med. (1996) 12:635–58. 10.1016/S0749-0690(18)30193-9 8890108

[39] Hatch J Gill-Body KM Portney LG. Determinants of balance confidence in community-dwelling elderly people. Phys Ther. (2003) 83:1072–9. 10.1093/ptj/83.12.1072 14640866

[40] Berg K Wood-Dauphinee S Williams JI Gayton D. Measuring balance in the elderly: preliminary development of an instrument. Physiother Canada. (1989) 41:304–11. 10.3138/ptc.41.6.304

[41] Zecevic AA Salmoni AW Speechley M Vandervoort AA. Defining a fall and reasons for falling: comparisons among the views of seniors, health care providers, and the research literature. Gerontologist. (2006) 46:367–76. 10.1093/geront/46.3.367 16731875

[42] Covinsky KE Kahana E Kercher K Schumacher JG Kahana B Justice AC. History and mobility exam index to identify community-dwelling elderly persons at risk of falling. J Gerontol Series A Biol Sci Med Sci. (2001) 56:M253–9. 10.1093/gerona/56.4.M253 11283200

[43] Ghazi HF Elnajeh M Abdalqader MA Baobaid MF Rosli NS Syahiman N. The prevalence of falls and its associated factors among elderly living in old folks home in Kuala Lumpur, Malaysia. Int J Commun Med Public Health. (2017) 4:3524–9. 10.18203/2394-6040.ijcmph20174214

[44] Rizawati M Mas AS. Home environment and fall at home among the elderly in Masjid Tanah Province. J Health Transl Med. (2008) 11:72–82. 10.22452/jummec.vol11no2.6

[45] Cruz DT Leite IC. Falls and associated factors among elderly persons residing in the community. Revista Brasileira de Geriatria e Gerontologia. (2018) 21:532–41. 10.1590/1981-22562018021.180034

[46] Yeong UY Tan SY Yap JF Choo WY. Prevalence of falls among community-dwelling elderly and its associated factors: a cross-sectional study in Perak, Malaysia. Malays Fam Phys. (2016) 11:7. 28461842 PMC5405326

[47] Hajek A König HH. Falls and subjective well-being. Results of the population-based German ageing survey. Arch Gerontol Geriatr. (2017) 72:181–6. 10.1016/j.archger.2017.06.010 28692833

[48] Azidah AK Hasniza H Zunaina E. Prevalence of falls and its associated factors among elderly diabetes in a tertiary center, Malaysia. Curr Gerontol Geriatr Res. (2012) 5:539073. 10.1155/2012/539073 22693496 PMC3369479

[49] Deandrea S Lucenteforte E Bravi F Foschi R La Vecchia C Negri E. Risk factors for falls in community-dwelling older people: a systematic review and meta-analysis. Epidemiology. (2010) 21:658–68. 10.1097/EDE.0b013e3181e89905 20585256

[50] Grundstrom AC Guse CE Layde PM. Risk factors for falls and fall-related injuries in adults 85 years of age and older. Arch Gerontol Geriatr. (2012) 54:421–8. 10.1016/j.archger.2011.06.008 21862143 PMC3236252

[51] Sai AJ Gallagher JC Smith LM Logsdon S. Fall predictors in the community dwelling elderly: a cross sectional and prospective cohort study. J Musculoskelet Neuronal Interact. (2010) 10:142–50. 20516631

[52] Bogle Thorbahn LD Newton RA. Use of the berg balance test to predict falls in elderly persons. Phys Ther. (1996) 76:576–83. 10.1093/ptj/76.6.576 8650273

[53] Robbins AS Rubenstein LZ Josephson KR Schulman BL Osterweil D Fine G. Predictors of falls results of two. Arch Int Med. (1989) 149:1628–33. 10.1001/archinte.1989.00390070138022 2742437

[54] Yip YB Cumming RG. The association between medications and falls in Australian nursing-home residents. Med J Aust. (1994) 160:14–8. 10.5694/j.1326-5377.1994.tb138194.x 7903790

[55] Cebolla EC Rodacki ALF Bento PCB. Balance, gait, functionality and strength: comparison between elderly fallers and non-fallers. Braz J Phys Ther. (2015) 19:146–51. 10.1590/bjpt-rbf.2014.0085 25993628 PMC4481835

[56] Borel L Alescio-Lautier B. Posture and cognition in the elderly: interaction and contribution to the rehabilitation strategies. Neurophys Clin. (2014) 44:95–107. 10.1016/j.neucli.2013.10.129 24502910

[57] Wong CY. Exploring the relationship between mobile phone and senior citizens: a Malaysian perspective. Int J Hum Comput Interact. (2011) 2:65–77.

[58] Petrescu-Prahova M Belza B Kohn M Miyawaki C. Implementation and maintenance of a community-based older adult physical activity program. Gerontologist. (2016) 56:677–86. 10.1093/geront/gnv024 26035891 PMC6282690

[59] Sudsawad P. Knowledge Translation: Introduction to Models, Strategies and Measures. Austin, TX: Southwest Educational Development Laboratory, National Center for the Dissemination of Disability Research (2007). p. 5–9.

[60] Vaportzis E Giatsi Clausen M Gow AJ. Older adults perceptions of technology and barriers to interacting with tablet computers: a focus group study. Front Psychol. (2017) 8:1687. 10.3389/fpsyg.2017.01687 29071004 PMC5649151

[61] Sin AK Ahmad A Zaman HB Sulaiman R. A wearable device for the elderly: a case study in Malaysia. In: Proceedings of the 6th International Conference on Information Technology and Multimedia; 2014 Nov 18. Putrajaya: IEEE (2014). p. 318–23.

[62] Chen K Chan AH. Use or non-use of gerontechnology—A qualitative study. Int J Environ Res Public Health. (2013) 10:4645–66. 10.3390/ijerph10104645 24084674 PMC3823313

[63] Lam JCY Lee MKO. Digital inclusiveness—Longitudinal study of internet adoption by older adults. J Manag Inf Syst. (2006) 22:177–206. 10.2753/MIS0742-1222220407

[64] Lagana L. Enhancing the attitudes and self-efficacy of older adults toward computers and the Internet: results of a pilot study. Educ Gerontol. (2008) 34:831–43. 10.1080/03601270802243713 20148185 PMC2817993

[65] Peek STM Luijkx KG Vrijhoef HJM Nieboer ME Aarts S Van Der Voort CS. Origins and consequences of technology acquirement by independent-living seniors: towards an integrative model. BMC Geriatrics. (2017) 17:1–18. 10.1186/s12877-017-0582-5 28830444 PMC5567629

